# Cannabinoid hyperemesis syndrome and cannabis withdrawal syndrome: a review of the management of cannabis-related syndrome in the emergency department

**DOI:** 10.1186/s12245-022-00446-0

**Published:** 2022-09-08

**Authors:** Mohammad Razban, Aristomenis K. Exadaktylos, Vincent Della Santa, Eric P. Heymann

**Affiliations:** 1 Department of Internal Medicine, University Hospital of Geneva, Geneva, Switzerland; 2 University of Bern, Bern, Switzerland; 3 Department of Emergency Medicine, University Hospital of Bern, Bern, Switzerland; 4 Department of Emergency Medicine, Cantonal Hospital of Neuchatel, Neuchatel, Switzerland

**Keywords:** Cannabis, Hyperemesis, Withdrawal, Syndrome, Treatments

## Abstract

**Background:**

Cannabis-related medical consultations are increasing worldwide, a non-negligible public health issue; patients presenting to acute care traditionally complain of abdominal pain and vomiting. Often recurrent, these frequent consultations add to the congestion of already chronically saturated emergency department(s) (ED). In order to curb this phenomenon, a specific approach for these patients is key, to enable appropriate treatment and long-term follow-up.

**Objectives:**

This study reviews cannabinoid hyperemesis syndrome (CHS) and cannabis withdrawal syndrome (CWS), in a bid to help promote better understanding and handling of pathologies associated with chronic cannabis use. Following a literature review, we present a novel therapeutic algorithm aimed at guiding clinicians, in a bid to improve long-term outcomes and prevent recurrences.

**Methods:**

Using the keywords “Cannabis,” “Hyperemesis,” “Syndrome,” “Withdrawal,” and “Emergency Medicine,” we completed a literature review of three different electronic databases (PubMed®, Google scholar®, and Cochrane®), up to November 2021.

**Results:**

Although often presenting with similar symptoms such as abdominal pain and vomiting, cannabinoid hyperemesis syndrome (CHS) and cannabis withdrawal syndrome (CWS) are the result of two differing pathophysiological processes. Distinguishing between these two syndromes is essential to provide appropriate symptomatic options.

**Conclusion:**

The correct identification of the underlying cannabis-related syndrome, and subsequent therapeutic choice, may help decrease ED presentations. Our study emphasizes the importance of both acute care and long-term outpatient follow-up, as key processes in cannabis-related disorder treatment.

## **Background**

The use of cannabis as a recreational substance has increased worldwide in the past 20 years, as its use becomes more socially accepted. Now regularly consumed by a large spectrum of the population, this trend has resulted in an increase in the number of cannabis-related medical consultations [1], making its consumption a non-negligible public health issue. In Switzerland, nearly 1/3 of the population over the age of 15 years has already tried cannabis for reasons other than medical purposes [2].

A well-recognized association of symptoms, abdominal pain, and vomiting is, in chronic users, generally attributed to cannabinoid hyperemesis syndrome (CHS). They are however also encountered in cannabis withdrawal syndrome (CWS), an often debated but officially (ICD and DSM) recognized withdrawal syndrome. Distinguishing between these two pathologies is important as the underlying mechanism and treatment options differ.

This study aims to present pathophysiological differences in a bid to help guide physician therapeutic decisions and optimize long-term patient outcomes.

## Methods

Using the keywords “Cannabis,” “Hyperemesis,” “Syndrome,” “Withdrawal,” and “Emergency Medicine,” we performed an in-depth literature review of 3 electronic databases (PubMed®, Google scholar®, and Cochrane®), aimed at all articles containing any of the above keywords, until November 2021.

**Fig. 1:**
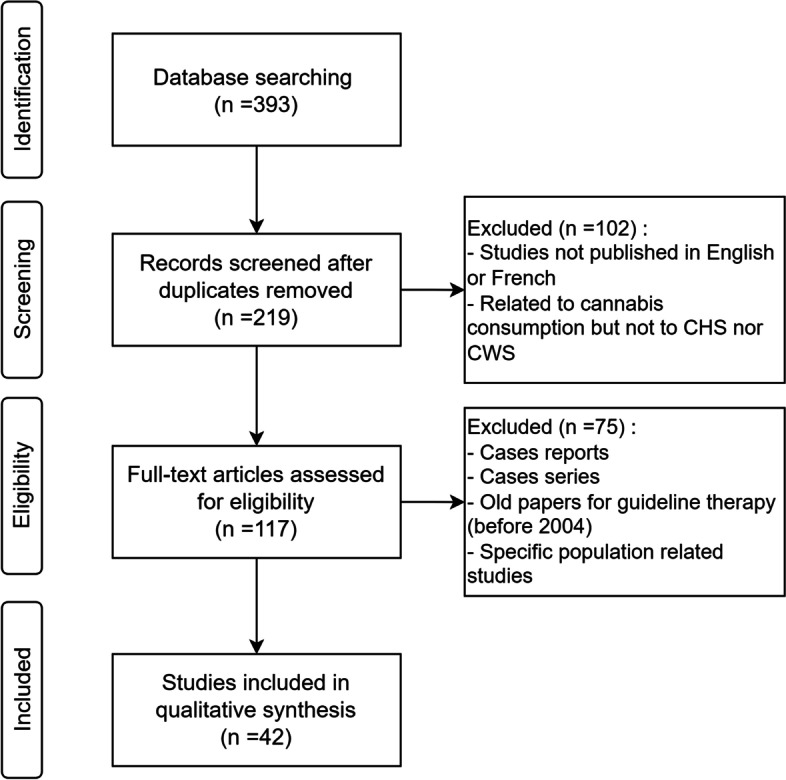
Literature selection flowchart

All levels of evidence were considered and reviewed. Publications in English and in French and full-text articles were considered. Three hundred ninety-three publications were identified, of which 42 were included for our study; we did not include individual case reports as these are associated with a low level of evidence, although we did consider one case series which included 98 patients, in light of the number of patients in the publication. Systematic reviews, randomized controlled trials, retrospective studies, and meta-analysis from 1998 to 2021 were included. Figure 1 presents the literature review selection and retention process.

## Pathophysiology of abdominal pain and emesis

First identified in 1990, the innate endocannabinoid system serves multiple endocrine functions, through the activation of receptors (principally CB1 and CB2) within the central nervous system, as well as in bones, the gastrointestinal tract, hepatocytes, pancreatic cells, muscles, uterus, and adipose cells. When exposed to low doses of cannabinoid, the activation of this system principally has an antiemetic effect. Within the gastrointestinal tract, this interaction inhibits the opening of the gastro-esophageal sphincter, slows peristalsis (through its action on smooth muscle cells), and lowers acid secretion [3, 4]. In the CNS, activation of these receptors has a direct role in regulating the sympathetic and hypothalamic-pituitary-adrenal axis, preventing overstimulation.

Working in a dose-dependent and biphasic manner, progressive desensitization of CB1 receptors can occur when overstimulated, creating paradoxical effects. This overstimulation is thought to be multifactorial and relates in part to the lipophilic properties of cannabinoids, which store in body fat and are subsequently released in chronic users (whose reserves are elevated from continuous intake) [5, 6]. The fact that not all chronic users develop CHS suggests genetic predisposition, a feature recently described, via the identification of five gene mutations which seem to confer some level of protection from paradoxical effects of overstimulation of the endocannabinoid system [7].

In patients predisposed to symptoms, overstimulation of said receptors may result in increased gastric acid secretion and impaired gut motility and relaxation of the gastro-esophageal sphincter, as well as dysregulated basal sympathetic activity, altogether resulting in hyperemesis. Further to this, abdominal discomfort can also occur and is thought to be due to THC-induced splanchnic vasodilation (and cutaneous vasoconstriction), a phenomenon known as “cutaneous-stealing syndrome” [8]. The excessive self-administration of hot showers, a feature well-described in the literature [5], is thought to reverse this by inducing peripheral vasodilatation of the peripheries and redistributing blood flow away from the gastrointestinal tract [4]. Interaction of CB receptors with the TRPV1 receptor explains further this phenomenon and the therapeutic effect of capsaicin cream (see below), with high doses of cannabinoids causing hypothermia and low doses of hyperthermia [9].

In addition, chronic cannabis users often develop symptoms of addiction. One suggested reason is via the stimulation of the dopaminergic system by cannabinoids in the nucleus accumbens. While understimulation of this system leads to vomiting and abdominal pain, this reward pathway can also cause paradoxical effects when overstimulated, such as anhedonia and lower positive and higher negative emotionality scores [10], all symptoms regularly seen in chronic users. Sudden abstinence or drop in intake in patients whose dopaminergic pathway has been upregulated in response to high doses of cannabinoids could therefore theoretically cause symptoms of withdrawal. This has been well demonstrated in regularly THC-exposed animals and humans, whom, when administered Rimonabant© (a CB1 receptor blocker, used to treat obesity) [11, 12], rapidly develop abdominal pain and hyperemesis. In addition to these withdrawal symptoms, other disturbances have also been described in these patients, such as irritability, anxiety, sleep disturbances, and loss of appetite. It is believed these occur through decreased mesolimbic dopamine function [13].

Thus, chronic users seem to develop symptoms from stimulation of already overstimulated CB receptors (CHS) but can also develop symptoms upon cessation through decreased central nervous system stimulation (CWS). As mechanisms vary, correctly identifying which syndrome a chronic THC user is presenting is key to choosing the appropriate treatment.

## Differentiating CHS from CWS

Bearing CHS and CWS in mind, patients who are chronic THC users presenting with hyperemesis and abdominal pain can have a multitude of other pathologies; Table 1 presents a non-exhaustive list of differential diagnoses illustrating the wide range of possibilities.

**Table 1:** Non-exhaustive list of differential diagnosis of hyperemesis

Pregnancy	
Esophageal motility disorder	
Bulimia/anorexia	
Choledocholithiasis	
Cyclic vomiting syndrome	
Pancreatitis	
Intoxication (accidental or deliberate)	
Gastritis	
Esophagitis	
Inflammatory bowel disease	
Lead poisoning	
Sickle cell anemia	
Acute intermittent porphyria	

## Clinical history

As with any patient, obtaining a detailed history is a vital component to correctly establish a differential diagnosis. In terms of abdominal pain and hyperemesis, this relies on excluding a wide range of differentials (see Table 1) thanks to a thorough exam and the use of paraclinical tests (see below), and focusing on certain differences may help differentiate CHS from CWS.

Epidemiologically, CHS occurs predominantly in males, whereas CWS affects women more frequently. A correlation between the time of onset of symptoms and last consumption has also been demonstrated, with CHS symptoms usually appearing within 24 h of last consumption (whereas CWS can occur anytime from 1 day to 10 days after last consumption). Patients presenting > 1 day after intake of THC are thus more likely to be suffering from CWS than CHS [14]. In terms of symptoms, the combination of abdominal pain and vomiting occurs more frequently in CHS than CWS (85.1% versus 8.3%), though this is unspecific. Hot showers, for reasons described above, exclude the diagnosis of CWS, being present in 92.3% of patients with CHS (versus 0% in CWS) [14, 15]. Psychological symptoms such as irritability, sleep difficulty, nervousness, restlessness, and depression tend to affect CWS patients more frequently, potentially significantly impairing daily life [14].

In terms of clinical course, patients suffering from CHS often describe three clear phases, which occur after a period of regular cannabis consumption, during which doses have been continuously increased to obtain desired effects (tolerance) [15]. An initial phase of morning sickness and dyspepsia, lasting anywhere from several months to years, is usually the prodromal phase, during which patients generally tend to increase their consumption to benefit from its antiemetic effect. This is then often followed by a second phase where patients experience recurrent episodes of hyperemesis with abdominal pain occurring in the hours after THC consumption. Each acute episode can last up from 24 to 48 h, and it is during this phase that patients are most likely to take hot showers for symptomatic relief and tend to present to the ED. Following this second phase, a recovery phase with two possible outcomes arises: either the patient continues THC consumption, in which case the symptoms appear again (as do the number of ED visits), or they will completely abstain, in which case, if said abstinence is maintained, CHS symptoms do not recur [4, 5, 16].

CWS, on the other hand, tends to present in chronic users within 1–10 days after last THC intake, with a peak incidence between days 2 and 6. No correlation has been established between symptoms severity and quantity (of THC) previously consumed, and initial presentation (to acute care) tends to vary, with a clinical course not well defined. Symptoms, which include nausea and vomiting as well as psychological and other somatic issues, generally worsen the further the patient is from last consumption, and can last up to 4 weeks. This likely corresponds to the time needed for CB1 receptors to return to their original state in the central dopaminergic pathways; this important feature is key to long-term management of these patients, who require ambulatory follow-up rather than simple symptomatic relief [13]. Table 2 summarizes the relevant clinical differences.

**Table 2:** Key clinical history information

	Cannabinoid hyperemesis syndrome	Cannabis withdrawal syndrome
Onset of symptoms, from last consumption of cannabis	< 24 h	> 24 h
Compulsive hot showers, as symptomatic relief	Yes	No
Accompanying psychological symptoms	No	Yes
Clinical course/pattern	Well described; 3 phases; development of tolerance with escalating dosing	No
Quantity correlating with severity	No	Yes
Relief	Symptoms worsened by cannabis consumption	Symptoms relieved by cannabis consumption

## Paraclinical examination

While recurrent presentations may carry a bias, the initial presentation of a patient with hyperemesis and/or abdominal is usually associated with a wide range of paraclinical tests ordered, in light of the many differential diagnoses possible. A detailed history and clinical examination often renders these additional investigations obsolete, with little yielded benefit. Nonetheless, the severity of presentation may warrant a blood workup to exclude complications of vomiting-induced dehydration and dyselectrolytemia. An ECG is also generally advisable to exclude electrophysiological abnormalities (corrected QT interval, QRS interval, etc.), in light of differential diagnoses (e.g., inferior ST-Elevation Myocardial Infarct) and to anticipate for potential QTc modifying treatments [17]. Unfortunately, in addition to these tests, many patients undergo more invasive diagnostic tests such as CT, endoscopy, and occasionally exploratory laparoscopy, which often fail to find an alternative cause and expose the patient to potential complications [15, 18]. Screening of genetic predispositions may also provide some benefit in differential diagnosing, though this would not be of use in the acute setting; as of publication, 5 mutations have been identified as potentiators of CHS (COMT, TRPV1, CYP2C9, DRD2, and ABCA1) [7].

## Official classification

Since the early 2000s, both CHS and CWS have been recognized by the ICD-10 (F12.241 and F12.30 of the 10th edition of the International Classification of Diseases, respectively). CHS is also included in the Rome IV definition as a functional gastrointestinal disorder, while CWS is encompassed in the DSM-5 (5th edition of the Diagnostic and Statistical Manual of Mental Disorders) [19, 20].

For CHS, cardinal symptoms are cyclic vomiting accompanied by abdominal pain following cannabis consumption. These symptoms can be alleviated by hot showers, and complete resolution of the syndrome requires complete abstinence [15]. For CWS, patients should at least have three DSM-5 symptoms, within 1 week of complete cessation or reduction in cannabis use; this should occur following a heavy or prolonged use. Symptoms include loss of appetite, hypothymia, irritability, restlessness, anxiety, and sleep disturbance. While no consensus exists pertaining to the minimal duration of exposure, one study demonstrated that smoking ≥ 6 marijuana joints/day over 12 months triples the odds of CWS (in comparison to smoking 1 joint/day over the same period) [13, 21]. It is of interest to note that abdominal pain and vomiting are not included in the diagnostic criteria for the DSM-5; this further reflects the importance of a thorough medical history in establishing a diagnosis.

To fulfill diagnostic criteria, in addition to the above conditions, in both cases, symptoms should not be attributable to another medical condition or mental disorder and should significantly impede the performance of everyday activities [13, 22]. A summary of validated criterion for CHS and CWS are listed in Table 3.

**Table 3:** Diagnostic criteria of CHS and CWS

*CHS diagnostic criteria (Rome IV)*	*CWS diagnostic criteria (DSM-5)*
*Criteria fulfilled for the last 3 months with symptom onset at least 6 months before diagnosis*	*At least three criteria within one week of reducing or ceasing cannabis use should be present*
Stereotypical episodic vomiting resembling cyclic vomiting syndrome (CVS) in terms of onset, duration, and frequency Presentation after prolonged, excessive cannabis use Relief of vomiting episodes by sustained cessation of cannabis use	Irritability; anger or aggression Nervousness or anxiety Sleep difficulty Decreased appetite or weight loss Restlessness Depressed mood Somatic symptoms causing significant discomfort
*Supportive feature:* *May be associated with pathologic bathing behavior (prolonged hot baths or showers)*	

## Therapeutic approach

Patients presenting with hyperemesis and abdominal pain should be thoroughly examined, the potential for dehydration, prerenal acute kidney injury, and dyselectrolytemia being on the initial preoccupations for acute care. With the added advantage that hydration and electrolyte substitution may reduce symptomatology [15], the next step is focusing on specifically addressing nausea and hyperemesis. In light of differing pathological processes, the choice of agent should be tailored to the suspected diagnosis, hence the importance of obtaining a thorough medical history.

- Cannabinoid hyperemesis syndrome

Conventional antiemetics, such as metoclopramide, ondansetron, or domperidone, seem to have little to no effect in CHS [23, 24]. Benzodiazepines (mostly lorazepam) seem to be effective, but the level of evidence associated with their use is low [23]. Many other agents have been tested (ex. antidepressants, opioids, neuroleptics), the most promising being haloperidol (0.05–0.1 mg/kg IV/IM) and droperidol (0.625 mg IV/IM) [25, 26]. Black box warning of long QT syndrome associated with butyrophenone neuroleptics should not prevent their use for CHS, in light of the doses used (see below) [27]; a pre-administration ECG is nonetheless advisable. In addition to its antiemetic properties, this pharmaceutical class has the added advantage of reducing agitation, which may also be present in CHS patients presenting to acute care.

If present, abdominal pain should be addressed with capsaicin. It is the active component that give chilies their spice; this molecule seems particularly effective in treating abdominal pain in CHS, in contrast to more conventional treatments (paracetamol, NSAIDs, etc.) [24] or opioids, which may worsen nausea and vomiting and have the potential to create dependency and increase the number of ED consultations in search of opioids [16, 24]. Through its interaction with TRPV1 receptors, topical capsaicin, applied to the forearms and abdomen, combats hypothermia and helps redistributing blood flow away from the gastrointestinal tract (see above) [9, 28, 29]. In addition, the use of a PPI reduces the risk of esophageal and gastric mucosal lesions, following excessive vomiting [19]. Hot showers, if available, should also be offered as this may help reduce anxiety and will help guide your diagnosis (as pathognomonic of CHS). Finally, the only known treatment for CHS is long-term abstinence [15]. Table 4 summarizes the studies relevant to therapeutic options for CHS.

b. Cannabis withdrawal syndrome

**Table 4:** Cannabinoid hyperemesis syndrome study treatment

Study name	Study type and design	Treatment/intervention	Level of evidence*	Conclusion
2021 Pourmand A [28].	Retrospective—systematic review and meta-analysis	- Topical capsaicin 3–4 times a day	2a	- Low adverse effects - Meantime to response 325 min (5.41 h) and mean time to discharge 379 min (6.31 h)
2020 Ruberto J [25].	Randomized, controlled trial	- Haloperidol IV 0.05–0.1 mg/kg vs ondansetron IV 8mg	1b	- Haloperidol is superior to ondansetron for reducing abdominal pain, nausea/vomiting at 2 h after treatment - Discharge time is also shorter with haloperidol than ondansetron (3.1 h vs 5.6 h) - Four return visits with haloperidol treatment vs 6 with ondansetron
2019 McConachie M [29].	Retrospective—systematic review	- Topical capsaicin 3–4 times a day	2a	- In 2019, studies are of low methodological quality to assess capsaicin efficacy in CHS but the favorable benefit-risk balance makes it a reasonable treatment option
2019 Carl Lee [26]	Retrospective—cohort study	- Droperidol 0.625 mg IV used most of the time	2b	- Droperidol IV to treat nausea and vomiting in CHS significantly reduced length of stay (6.7 vs. 13.9 h) compared to the no droperidol treatment group - Median time to discharge after final drug administration was also shorter (137 min vs 185 min) - Overall use of antiemetic was less in the droperidol group
2017 Sorensen J[15].	Retrospective—systematic review	- Abstinence	3a	- Abstinence is the only definitive treatment identified for CHS

*OCEBM Oxford Center of Evidence-Based Medicine

CWS therapeutic options are more limited. Withdrawal symptoms seem to respond well to oral THC substitution [30], the concept being that these will interact with CB1 receptors, in a bid to counter severe symptoms due to rapid downregulation from withdrawal. Protocols using oral THC [31, 32], dronabinol [33], or nabiximols [34] have shown an improvement of withdrawal symptoms (altered mood and sleep, nausea and craving) and an increase in prolonged abstinence. Nabilone in association with zolpidem has also shown promising results [35]. Access to medical THC is however limited, and many countries (including Switzerland) have made access to these drugs very difficult, if not impossible. Alternative therapeutic options such as gabapentin [36] and behavioral therapies such as the twelve-step facilitation method (TFM), cognitive behavioral therapy (CBT), motivational enhancement therapy (MET), and contingency management (CM) [37, 38] (see Table 5) have all been shown to help improve symptoms while reducing relapse and should be sought after in countries where THC therapies are not yet available.

**Table 5:** Cannabis withdrawal syndrome study treatment

Study name	Study type and design	Treatment/intervention	Level of evidence*	Conclusion
2018 Zvolensky J [39].	Retrospective—cohort study	- Cannabis use problem, withdrawal symptoms, and self-efficacy for quitting	2b	- The difficulties in quitting cannabis consumption are related to greater withdrawal symptoms, more cannabis use problems, and lower self-efficacy for quitting
2016 John F [37].	Prospective—cohort study	- Twelve-step facilitation method (MET and CBT), marijuana anonymous meeting	1b	- Anonymous meetings improve abstinence in cannabis users - Twelve-step facilitation therapy decreases cannabis relapse and strengthens adherence to treatment
2016 Herrmann S [35].	Randomized, double-blind, placebo-controlled trial	- Zolpidem alone (12.5 mg) and zolpidem (12.5 mg) associated with nabilone (3 mg twice a day)	1b	- Zolpidem and nabilone each decrease cannabis withdrawal-related sleep disruption, but only a combination of both molecules alleviates global symptoms of withdrawal and decreased self-administration of active cannabis
2014 Irons G [40].	Prospective—cohort study	- Physical activity	1b	- Low level of physical activity is associated with a higher risk of relapse into cannabis consumption during the week following a quit attempt compared to a moderate/high level of physical activity
2014 Allsop J [34].	Double-blind randomized clinical inpatient trial	- 6-day regimen of nabiximols	1b	- Nabiximols improves cannabis withdrawal symptoms (cravings, irritability and depression) and abstinence in the short term but not in the long term
2011 Vandrey R [41].	Randomized, double-blind, placebo-controlled trial	- Zolpidem alone	1b	- Zolpidem alone can attenuate sleep disruption associated with cannabis withdrawal
2012 Mason J [36].	Randomized, double-blind, placebo-controlled trial	- Gabapentin 1200 mg/day	1b	- Gabapentin 1200 mg/day with an acceptable safety profile and no evidence of dependence has a significant effect on decreasing cannabis use and withdrawal symptoms
2011 Frances R [33].	Randomized, double-blind, placebo-controlled trial	- Dronabinol 20 mg twice a day for 8 weeks and tapered off over 2 weeks	1b	- Treatment retention was significantly higher and withdrawal symptoms were significantly lower on dronabinol than on placebo
2010 Budney J [38].	Systematic review	- CBT, MET, and CM	1a	- Behaviorally based interventions such as MET, CBT, and CM can help individuals to change their problematic use of cannabis
2007 Budney J [31].	Prospective—cohort study	- Daily doses of placebo, 30mg (10 mg/tid), or 90 mg (30 mg/tid) oral THC	1b	- In a dose-responsive manner oral THC reduces cannabis withdrawal symptoms
2004 Haney M [32].	Randomized, double-blind, placebo-controlled trial	- Daily oral THC capsules (10 mg)	1b	- Oral THC decreases symptoms and cravings associated with cannabis withdrawal (anxiety, misery, chills, self-reported sleep disturbance, anorexia, and weight loss).

*OCEBM Oxford Center of Evidence-Based Medicine

*CBT* cognitive behavioral therapy, *CM* contingency management, *MET* motivational enhancement therapy

No studies have evaluated the treatment of abdominal pain, as its incidence in CWS is significantly less than in CHS. Furthermore, in light of the pathophysiological processes behind CWS, its presence may not be a direct consequence to THC but simply a response to emesis (and if present, may be a sign that the patient is experiencing CHS rather than CWS). Thus, if present, it should theoretically be manageable with conventional non-opioid analgesics and anticholinergics (such as butylscopolamine), the latter having the advantage of increasing dopamine concentrations in the brain. Regarding nausea, antipsychotics should be withheld as they tend to decrease central dopamine levels and may worsen withdrawal symptoms such as craving [42]. Table 5 summarizes the most relevant studies and levels of proof.

iii. Abstinence and follow-up

In addition to abdominal pain, nausea, and vomiting, CHS and CWS are associated with many secondary issues, ranging from social isolation and financial difficulties (absence from work, cost of ED consults, water and electric bills in case of hot showers), as well as dermal burns from hot water in extreme cases of CHS [43].

In both syndromes, complete abstinence is the definitive treatment [15]. With a high potential for relapse (54% of patients achieving 2-week abstinence, and 71% relapse within 6 months [39]), follow-up of patients should be initiated, if possible, from acute care [39, 44]. This can be done through healthcare liaison officers, dedicated community outreach nurses, and/or group counseling sessions such as Marijuana Anonymous which works in a similar fashion to Alcoholics Anonymous, with sponsors and group discussions. These strategies have all demonstrated a reduction in relapse rates [13, 30, 37].

## Discussion

Like many EDs worldwide, the normalization of cannabis consumption has led to an increase in the number of cannabis-related consults in the ED (positive delta from 2.3 to 13.3 cases per 100,000 ED visits in the USA from 2006 to 2013) [43]. In light of the severity of their symptoms, these patients often require increased monitoring and accompaniment. With average ED times of 13.9 h [26], these patients, who often do not fill the criterion for hospitalization, are bound to already chronically oversaturated EDs and add to the pressure on healthcare systems. Worse, frequent ED consultations have been shown to lead to cognitive bias from teams, which could trivialize symptoms and result in missed alternative diagnoses [45]. In light of these factors, and based on the above literature review, we proceeded to review the management of chronic cannabis users presenting to our ED with hyperemesis, nausea, and/or abdominal pain. In a bid to share with other acute care units, we will now present our internal guidelines, reflecting the current level of evidence.

Upon arrival to our ED, chronic cannabis users presenting with hyperemesis and nausea are first triaged to exclude any other conditions or signs of shock. If hemodynamically stable, they are redirected to a consult area with access to a nearby shower. IV access is set up, with bloods taken (a minimum of creatinine, urea, electrolytes, liver function tests, lipase, and full blood count are sent) and crystalloid-based rehydration started, as patients are usually unable to drink water due to nausea and/or emesis. An ECG is usually rapidly done, and the patient is given a PPI (PO, if tolerated). A thorough medical history is then obtained, and depending on whether CHS or CWS is suspected (the request for a warm shower is interpreted by us as diagnostic of CHS), a trial therapy with either 0.625 mg droperidol (IV) or haloperidol 0.05–0.1 mg/kg (IV/IM) for CHS or ondansetron (4 mg IV) for CWS is initiated. While currently not readily available in the author’s country of practice (available usually as a magistral preparation), the addition of capsaicin cream in the acute ED treatment may be highly beneficial at this stage: not only will the administration be demonstrated; the efficacy may actually improve ambulatory use.

The patient is then observed, and, depending on symptom progression, a second dose of butyrophenone neuroleptics may be added. If abdominal pain is present, standard analgesics are prescribed if CWS is suspected (for CHS, the use of topical capsaicin can again only be encouraged). During their time in our ED, these patients are generally seen by our *frequent user (or complex case) team*. Composed of specialized community outreach nurses, the aim is to identify what support systems the patient has in place in the community but also helps, in case of recurrent visits, determine what triggers have led to renewed consumption, to enable ambulatory follow-up and promote long-term abstinence. They are also trained, and take the time, to explain the diagnosis (including the pathophysiological pathways) and, for CHS, discuss the risk of developing a withdrawal syndrome (CWS) [44], all while promoting complete abstinence.

Upon resolution of acute debilitating symptoms, patients are usually discharged with a prescription for a magistral preparation of capsaicin cream (0.075%) (application on forearms and abdomen, 3–4 times daily), as well as a PPI, for CHS [26, 27, 40]. For suspected CWS, the only oral THC available in Switzerland is nabiximols (Sativex©) and is currently only licensed for multiple sclerosis [46]. Patients are therefore usually prescribed non-opioid analgesia, a reserve for gabapentin, with or without zolpidem; though after a review by our emergency psychiatry team, some are having psychological-associated symptoms. As there is always a risk for a missed diagnosis, prior to discharge, patients are reminded to present to the nearest healthcare center should their condition deteriorate or new symptoms develop. Our EMR system has been modified to automatically flag these patients when they present to our hospital, to ensure that healthcare teams have access to the long-term treatment plan (in patient notes). We believe this also helps reduce bias, as reading the notes illustrates the complexity of these syndromes.

Finally, after discharge, each patient is followed-up by our community outreach nurses, who review psychological, somatic, and social aspects of care. Follow-up is generally done on the phone, though community outreach nurses may see the patients within a dedicated clinic, to review progress, discuss symptomatic treatments, and prevent recurrent consultation to the ED [12]. Counseling may sometimes be required, though programs such as Marijuana Anonymous are currently non-existent in Switzerland, and so this is usually done with group behavioral therapy [30, 37]. A regular follow-up with the family doctor is also encouraged, as is physical activity, as this has been associated with prolonged abstinence [38, 40]. Should symptoms not resolve, or hospitalization be required for reasons other than purely symptomatic treatment (e.g., inadequate housing, previous psychiatric comorbidities, multiple failed attempts to quit), inpatient management including treatment (pain, nausea or insomnia for example), and psychosocial follow-up is governed according to a set protocol that includes a combination of the above [13].

**Fig. 2:**
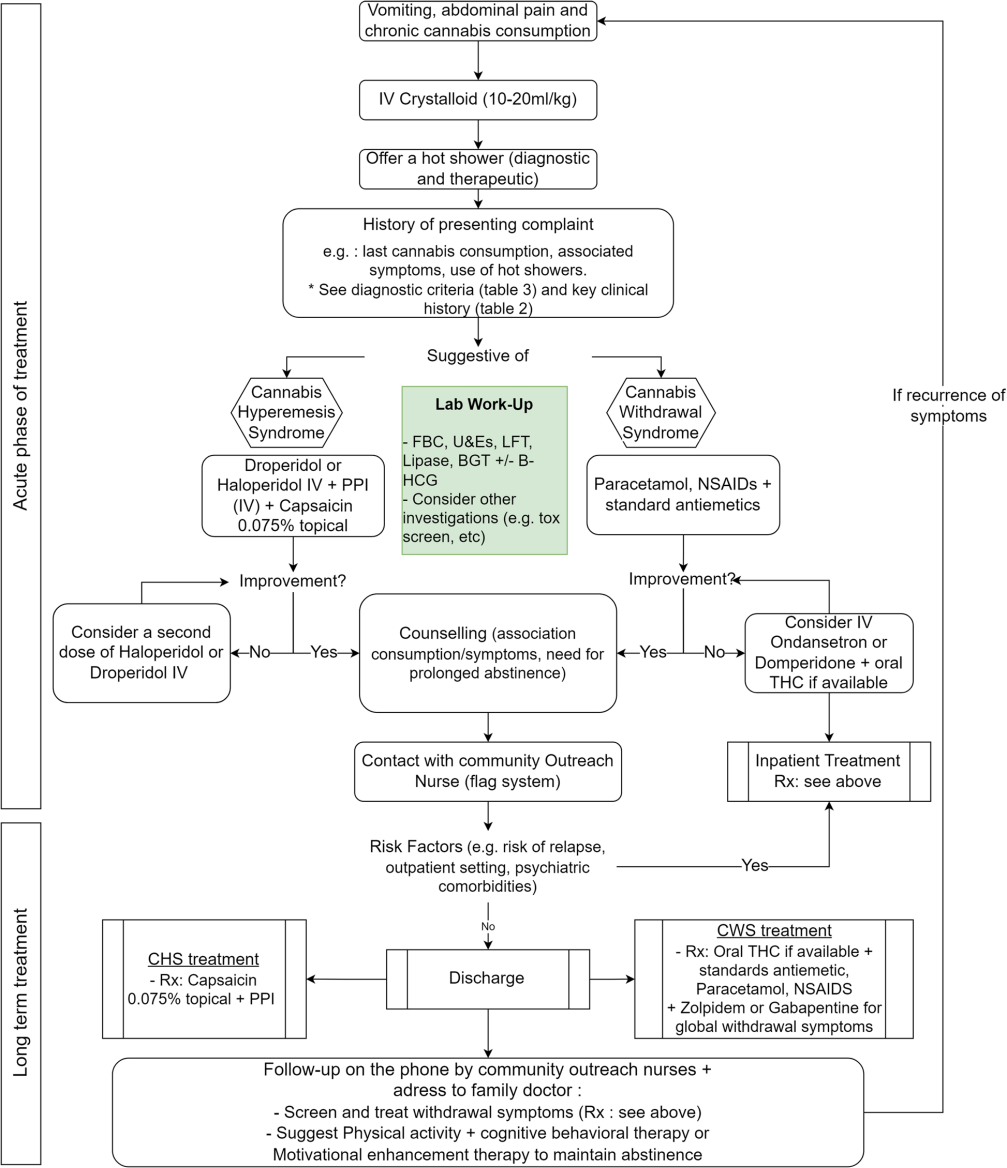
Proposed emergency department therapeutic algorithm for cannabis use disorder. Rx, recipe; FBC, full blood count; U&Es, urea and electrolytes blood test; LFT, liver function tests; BGT, blood glucose test; PPI, proton-pump inhibitors; MET, motivational enhancement therapy; CBT, cognitive behavioral therapy

Figure 2 summarizes our proposed emergency department therapeutic algorithm for cannabis use disorders.

## Limitations

While this paper covers many of the key pathophysiological and therapeutic possibilities for cannabis use disorders presenting to acute care, limitations arising from the retrospective nature of a literature review were identified during manuscript writing. We believe more research is needed regarding both acute and long-term treatment options.

## Conclusion

CHS and CWS are rapidly becoming major public health issues and add to the caseloads of already chronically overburdened ED. Optimization of ED care is possible but requires understanding the pathophysiological differences of each syndrome. With the only known treatment being abstinence and the high risk of relapse, it is important to rely not solely on acute care but also on long-term follow-up strategies. Though rare, hospitalization may sometimes be required; we believe this can be reduced by a combination of specific acute care treatment choice and optimal community support programs.

## Data Availability

All data generated or analyzed during this study are included in this published article.

## Abbreviations

CBT: Cognitive behavioral therapy
CHS: Cannabinoid hyperemesis syndrome
CM: Contingency management
CNS: Central nervous system
CVS: Cyclic vomiting syndrome
CWS: Cannabis withdrawal syndrome
ED: Emergency department
FBC: Full blood count
BGT: Blood glucose test
LFTs: Liver function tests
MET: Motivational enhancement therapy
PPI: Proton-pump inhibitors
Rx: *Recipe* (prescription)
THC: Tetrahydrocannabinol
TFM: Twelve-step facilitation method
U&Es: Urea and electrolytes blood test
